# Supplementary material for: Urinary N-acetylglucosaminidase in People Environmentally Exposed to Cadmium Is Minimally Related to Cadmium-Induced Nephron Destruction
Source: Toxics. 2024 Oct 25;12(11):775. doi: 10.3390/toxics12110775 (PMC11598048; doi:10.3390/toxics12110775)

Supplemental Material: Urinary N-acetylglucosaminidase in People Environmentally Exposed to Cadmium Is Minimally Related to Cadmium-Induced Nephron Destruction

Soisungwan Satarug

Table S1. The prevalence odds ratios for low eGFR in relation to tubular proteinuria assessed by β2M excretion normalized to creatinine excretion.

Independent Variables/Factors	Low eGFR				
	β Coefficients (SE)	POR	95% CI		p
			Lower	Upper	
Age, years	0.105 (0.023)	1.111	1.063	1.161	<0.000
BMI, kg/m^2	0.136 (0.050)	1.145	1.038	1.264	0.007
Log$_2$[(E_{Cd}/E_{cr}) × 10^3], μg/ g creatinine	0.218 (0.110, 3.930)	1.243	1.002	1.542	0.047
Gender	−0.547 (0.430)	0.579	0.249	1.345	0.204
Hypertension	0.771 (0.401)	2.162	0.985	4.746	0.055
Smoking	−0.197 (0.421)	0.821	0.360	1.872	0.639
$E_{β2M}/E_{cr}$, μg/ g creatinine					
>300	Referent				
300−1000	0.786 (0.570)	2.196	0.719	6.705	0.167
>1000	2.269 (0.514)	9.667	3.532	26.46	<0.001

POR, prevalence odds ratio; CI, confidence interval; β, regression coefficient; SE, standard error of mean; BMI, body mass index; eGFR, estimated glomerular filtration rate. Numbers of subjects in the $E_{β2M}/E_{cr}$ >300, 300−1000 and > 1000 μg/ g creatinine groups were 596, 45 and 39, respectively. For all tests, p-values ≤ 0.05 indicate a statistical significance.

Table S2. Predictors of the excretion of β2M normalized to creatinine excretion ($E_{β2M}/E_{cr}$).

Independent Variables/ Factors	Log$_{10}$[($E_{β2M}/E_{cr}$) ×10^3], μg/g creatinine							
	Males, n = 277		Females, n = 425		Low-Cd burden [a], n = 166		High-Cd burden, n = 536	
	β	p	β	p	β	p	β	p
Age, years	−0.147	0.032	0.018	0.714	0.086	0.490	0.045	0.219
BMI, kg/m^2	−0.151	0.001	−0.126	0.001	−0.130	0.122	−0.095	0.002
Log$_2$[(E_{Cd}/E_{cr}) × 10^3], μg/g creatinine	0.430	<0.001	0.486	<0.001	0.032	0.698	0.515	<0.001
eGFR, mL/min/1.73 m^2	−0.427	<0.001	−0.204	<0.001	0.042	0.705	−0.292	<0.001
Hypertension	−0.033	0.459	−0.017	0.659	−0.007	0.937	0.002	0.946
Smoking	0.026	0.550	0.039	0.307	0.125	0.171	0.029	0.359
Gender	−	−	−	−	0.064	0.521	−0.031	0.308
Adjusted R^2	0.516	<0.001	0.471	<0.001	−0.013	0.667	0.618	<0.001

n, number of subjects; eGFR, estimated glomerular filtration rate; β, standardized regression coefficient; BMI, body mass index; adjusted R^2, coefficient of determination. [a] Low and high burdens of Cd were indicated by E_{Cd}/C_{cr} <0.01 and ≥ 0.01 μg/L filtrate. β indicates strength of association of $E_{β2M}/E_{cr}$ with seven independent variables (first column). Adjusted R^2 indicates the

proportion of the variation of $E_{β2M}/E_{cr}$, which was explained by all independent variables. *p*-values ≤ 0.05 indicate statistically significant associations of independent variables with $E_{β2M}/E_{cr}$.

Table S3. The prevalence odds ratios for $β_2$-microglobilinuria in relation to tubular injury assessed by NAG excretion normalized to creatinine clearance.

Independent Variables/Factors	β2-microglobulinuria				
	β Coefficients (SE)	POR	95% CI Lower	95% CI Upper	*p*
Age, years	0.070 (0.014)	1.072	1.043	1.103	<0.001
BMI, kg/m²	−0.105 (0.037)	0.901	.837	.969	0.005
$Log_2[(E_{Cd}/C_{cr}) \times 10^5]$, µg/ L filtrate	0.533 (0.098)	1.705	1.406	2.066	<0.001
$Log_2[(E_{NAG}/C_{cr}) \times 10^3]$, U/ L filtrate	0.722 (0.134)	2.058	1.583	2.676	<0.001
Gender	0.908 (0.278)	2.480	1.438	4.280	0.001
Hypertension	0.068 (0.275)	1.070	.624	1.835	0.806
Smoking	0.184 (0.286)	1.202	.687	2.103	0.520

POR, prevalence odds ratio; CI, confidence interval; β, regression coefficient; SE, standard error of mean; BMI, body mass index; $β_2$-microglobilinuria was defined as $(E_{β2M}/C_{cr}) \times 100 \geq 300$ µg/L filtrate. For all tests, *p*-values ≤ 0.05 indicate a statistical significance.

Table S4. The prevalence odds ratios for $β_2$-microglobilinuria in relation to tubular injury assessed by NAG excretion normalized to creatinine excretion.

Independent Variables/Factors	β2-microglobulinuria				
	β Coefficients (SE)	POR	95% CI Lower	95% CI Upper	*p*
Age, years	0.060 (0.014)	1.062	1.032	1.092	<0.001
BMI, kg/m²	−0.068 (0.041)	0.934	0.861	1.012	0.097
$Log_2[(E_{Cd}/E_{cr}) \times 10^3]$, µg/g creatinine	0.324 (0.086)	1.382	1.168	1.634	<0.001
$Log_2[(E_{NAG}/E_{cr}) \times 10^3]$, U/g creatinine	0.883 (0.162)	2.417	1.758	3.323	<0.001
Gender	0.850 (0.312)	2.339	1.268	4.314	0.006
Hypertension	0.006 (0.335)	1.006	0.522	1.941	0.985
Smoking	0.104 (0.329)	1.109	0.581	2.116	0.753

POR, prevalence odds ratio; CI, confidence interval; β, regression coefficient; SE, standard error of mean; BMI, body mass index; $β_2$-microglobilinuria was defined as $E_{β2M}/E_{cr}) \geq 300$ µg/ g creatinine. For all tests, *p*-values ≤ 0.05 indicate a statistical significance.

Table S5. Predictors of the excretion of NAG normalized to creatinine excretion (E_{NAG}/E_{cr}.).

Independent Variables/ Factors	$Log_{10}[(E_{NAG}/E_{cr}) \times 10^3]$, U/g creatinine							
	Males, *n* = 277		Females, *n* = 427		Low-Cd burden [a], *n* = 186		High-Cd burden, *n* = 538	
	β	*p*	β	*p*	β	*p*	β	*p*
Age, years	−0.254	0.001	−0.057	0.294	−0.135	0.223	0.051	0.167
BMI, kg/m²	−0.098	0.056	−0.087	0.042	−0.022	0.766	−0.037	0.240
$Log_2[(E_{Cd}/E_{cr}) \times 10^3]$, µg/g creatinine	0.652	<0.001	0.520	<0.001	0.487	<0.001	0.638	<0.001
eGFR, mL/min/1.73 m²	−0.151	0.054	−0.100	0.059	0.063	0.520	−0.171	<0.001

Hypertension	0.041	0.414	−0.088	0.034	0.111	0.138	0.008	0.779
Smoking	−0.151	0.002	−0.042	0.323	−0.079	0.331	0.019	0.544
Gender	–	–	–	–	−0.007	0.941	0.029	0.354
Adjusted R^2	0.395	<0.001	0.350	<0.001	0.200	<0.001	0.608	<0.001

n, number of subjects; eGFR, estimated glomerular filtration rate; β, standardized regression coefficient; BMI, body mass index; adjusted R^2, coefficient of determination. [a] Low and high burdens of Cd were indicated by E_{Cd}/C_{cr} <0.01 and ≥ 0.01 μg/L filtrate. β indicates strength of association of E_{NAG}/E_{cr} with seven independent variables (first column). Adjusted R^2 indicates the proportion of the variation of E_{NAG}/E_{cr}, which was explained by all independent variables. *p*-values ≤ 0.05 indicate statistically significant associations of independent variables with E_{NAG}/E_{cr}.

Figure S1. Normal distribution of age in whole group and subgroups of men and women.

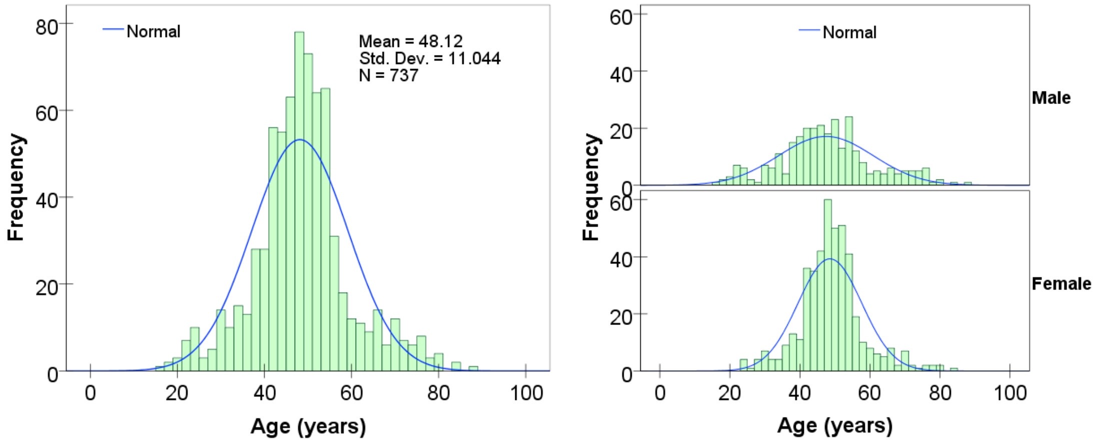

Figure S2. Left-skewed distribution of eGFR in whole group, and subgroups of men and women

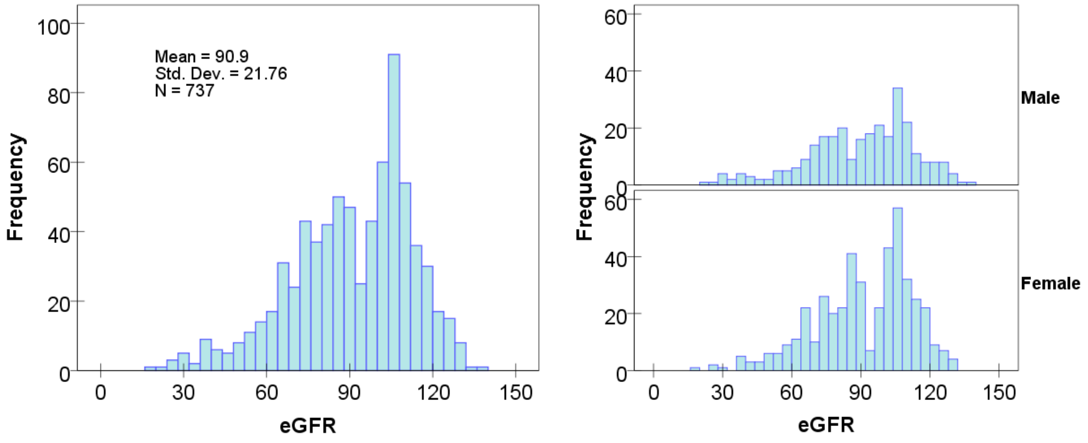

Figure S3. Distribution of E_{Cd}/C_{cr} and E_{NAG} /C_{cr} in whole group and subgroups of men and women

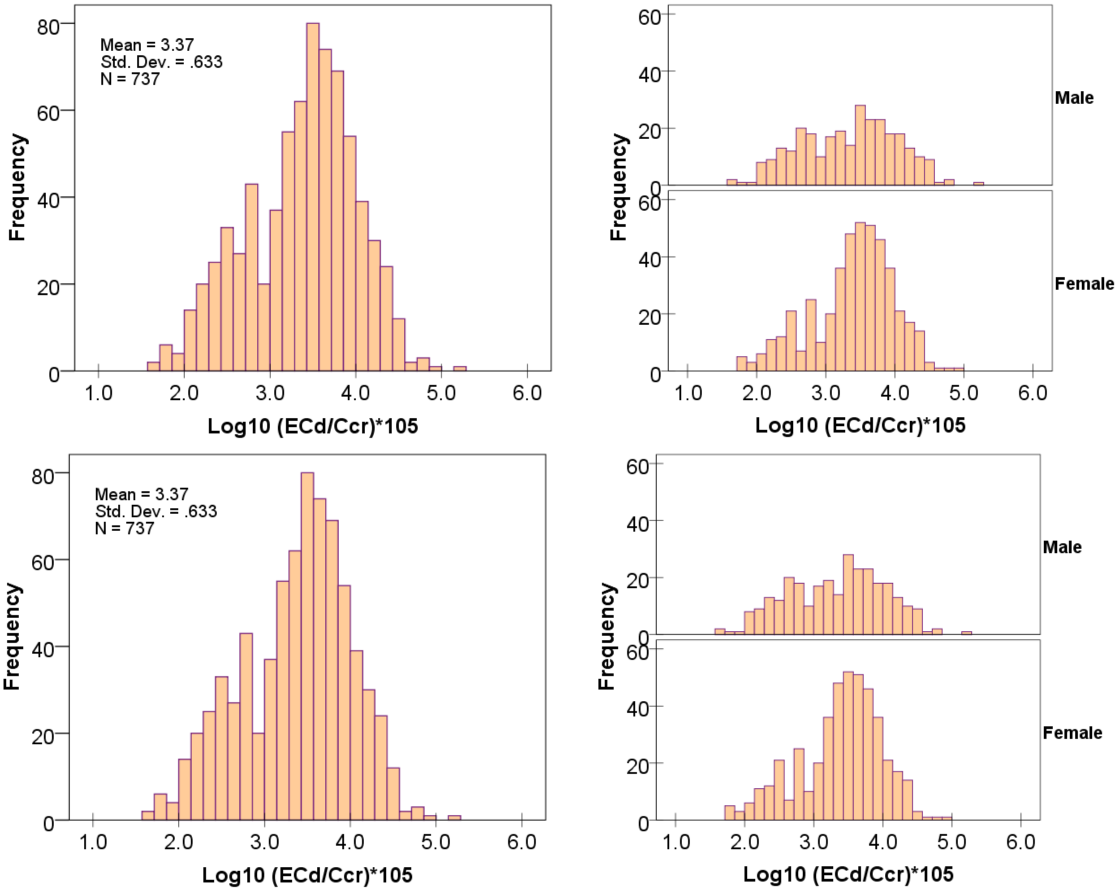

Figure S4. Distribution of E_{Cd}/E_{cr} and E_{NAG}/E_{cr} in whole group and subgroups of men and women

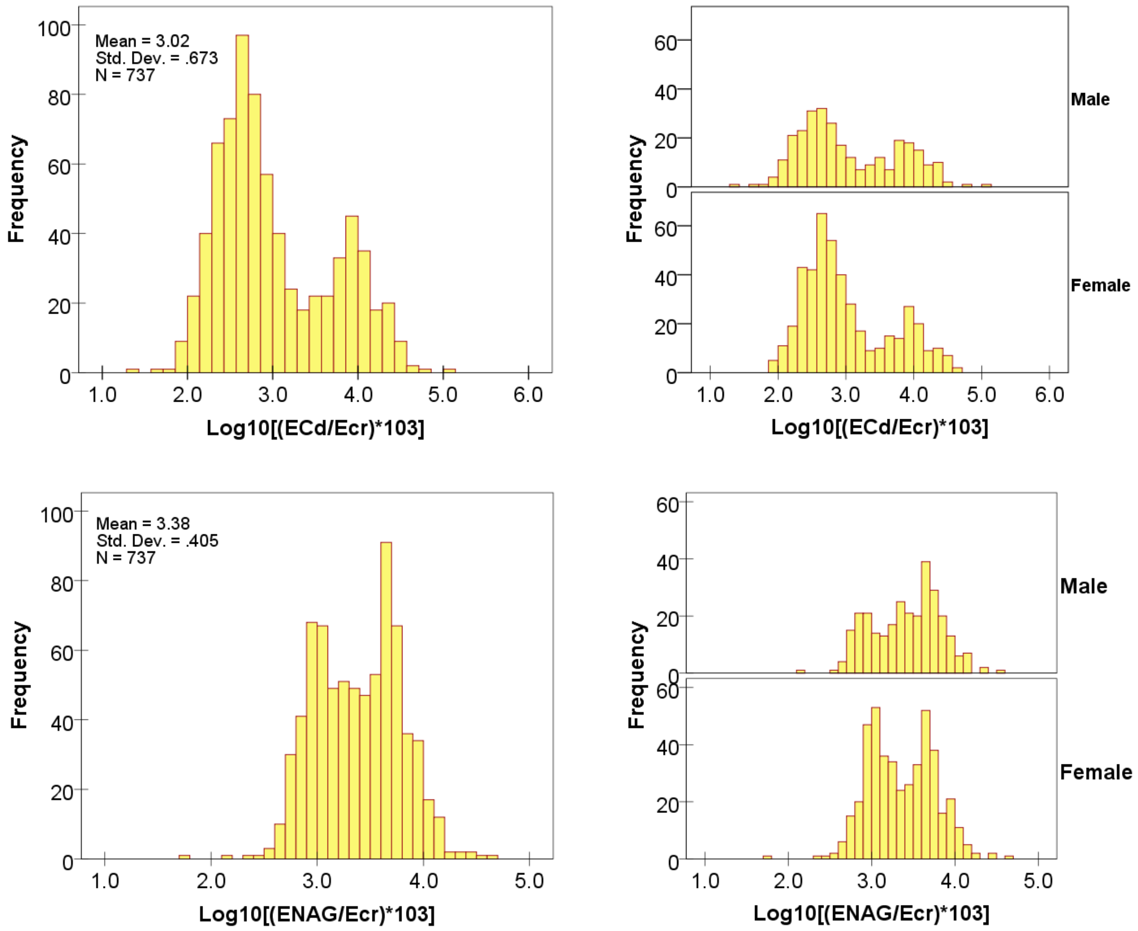

Figure S5. Comparing the distribution of $E_{\beta 2M}/C_{cr}$ and $E_{\beta 2M}/E_{cr}$ in whole group and subgroups of men and women

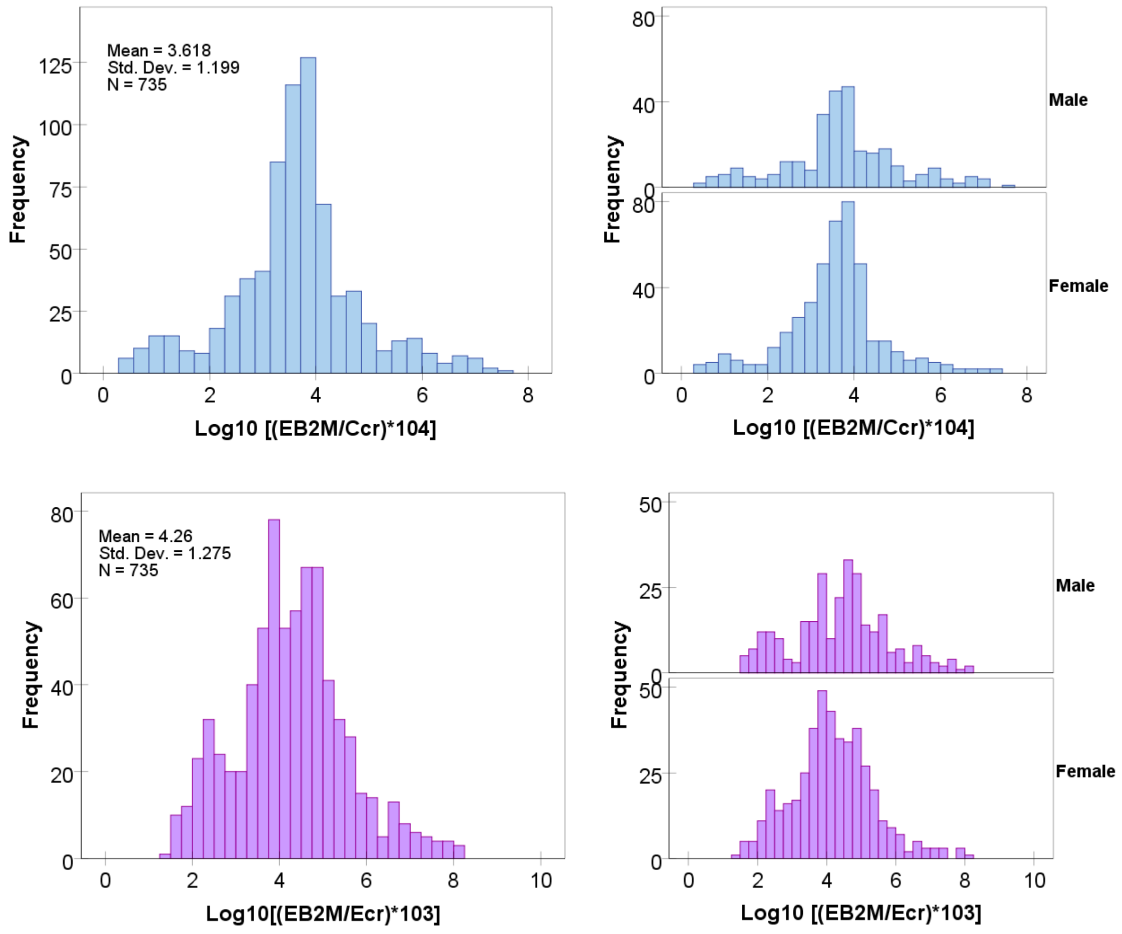

Supplement: Supplementary file 1 [file toxics-12-00775-s001.zip › toxics-3245342-supplementary.pdf]
